# Examination of Longitudinal Alterations in Alzheimer’s Disease-Related Neurogenesis in an APP/PS1 Transgenic Mouse Model, and the Effects of P33, a Putative Neuroprotective Agent Thereon

**DOI:** 10.3390/ijms231810364

**Published:** 2022-09-08

**Authors:** Titanilla Szögi, Emőke Borbély, Ildikó Schuster, Zsolt Bozsó, Miklós Sántha, Melinda E. Tóth, Botond Penke, Lívia Fülöp

**Affiliations:** 1 Department of Medical Chemistry, University of Szeged, Dóm tér 8, H-6720 Szeged, Hungary; 2 Institute of Biochemistry, Biological Research Centre, Temesvári Krt. 62., H-6726 Szeged, Hungary

**Keywords:** Alzheimer’s disease, neurogenesis, neuroinflammation, P33, APP/PS1 mice

## Abstract

Neurogenesis plays a crucial role in cognitive processes. During aging and in Alzheimer’s disease (AD), altered neurogenesis and neuroinflammation are evident both in C57BL/6J, APP_Swe_/PS1_dE9_ (Tg) mice and humans. AD pathology may slow down upon drug treatment, for example, in a previous study of our group P33, a putative neuroprotective agent was found to exert advantageous effects on the elevated levels of APP, Aβ, and neuroinflammation. In the present study, we aimed to examine longitudinal alterations in neurogenesis, neuroinflammation and AD pathology in a transgenic (Tg) mouse model, and assessed the putative beneficial effects of long-term P33 treatment on AD-specific neurological alterations. Hippocampal cell proliferation and differentiation were significantly reduced between 8 and 12 months of age. Regarding neuroinflammation, significantly elevated astrogliosis and microglial activation were observed in 6- to 7-month-old Tg animals. The amounts of the molecules involved in the amyloidogenic pathway were altered from 4 months of age in Tg animals. P33-treatment led to significantly increased neurogenesis in 9-month-old animals. Our data support the hypothesis that altered neurogenesis may be a consequence of AD pathology. Based on our findings in the transgenic animal model, early pharmacological treatment before the manifestation of AD symptoms might ameliorate neurological decline.

## 1. Introduction

In mammals, including humans, neurogenesis occurs throughout life. In adulthood, neuronal cell formation is present in the subventricular zone (SVZ) of the lateral ventricles and in the subgranular zone (SGZ) of the dentate gyrus (DG) within the hippocampus (HC) [1]. In both neurogenic niches, new cells are formed via mitosis from neural stem cells (NSCs), generating neural progenitor cells (NPCs) [2,3,4,5]. Some of the NPCs can migrate, proliferate and differentiate into neurons or glial cells, and these new cells can integrate into the circuitry of the olfactory bulb and the granular cell layer (GCL) of the DG, but a large proportion of these cells die by apoptosis early in their life cycle. Furthermore, both intrinsic modulators (signal transduction pathways, metabolic factors, hormonal stage, epigenetic factors as well as immune and vascular systems) and extrinsic factors (stress, drug and alcohol abuse, aging, physical activity, learning and environmental enrichments) affect the proliferation, differentiation, as well as the morphological and physiological maturation, fate determination and the survival of these new cells [5,6,7,8,9,10,11,12,13,14].

Adult hippocampal neurogenesis is essential for memory and learning processes. The extent of neurogenesis declines with age, and this is also made evident in neurodegenerative disorders such as Alzheimer’s disease (AD) [9,11,13,15,16,17]. AD is characterized by cognitive deficits, memory loss, altered neurogenesis and neuroinflammation. The familiar form of AD (FAD) is caused by mutations in genes that encode the amyloid-β protein precursor (APP), presenilin-1 (PS1) and presenilin-2 (PS2). In AD, APP can undergo several enzymatic cleavages by secretases. The metabolites produced in the amyloidogenic pathway (such as soluble APP (sAPP) and C99, the latter including APP’s intracellular domain (AICD) and amyloid-β (Aβ)), as well as those produced in the non-amyloidogenic pathway (sAPPα and C83, the latter including AICD and p3) have significant roles in the process of neurogenesis [9,14,17,18,19,20,21]. PS1 regulates the differentiation of NPCs. Furthermore, PS1 is the catalytic core of γ-secretase, which cleaves APP [9,13,14,17,18]. SAPPα is assumed to have neuroprotective effects: it presumably protects neurons, promotes neurogenesis and neurite outgrowth, thus modulating the proliferation and survival of NPCs [9,13,14,18,19,20,22,23]. Aβ may exert a negative effect on adult neurogenesis, as studies suggest a strong correlation between amyloidosis, Aβ deposition and impaired neurogenesis [16,18,19,22,24,25]. Declining neurogenesis may serve as a hallmark of AD pathology. It is also debated whether impaired neurogenesis is a contributing factor or a consequence of AD [26,27].

Aβ depositions and plaques are associated with neuroinflammation in AD. Microglia and astrocytes play a crucial role in AD pathology. In the early phase of the disease, microglia and astrocytes are activated and clustered around the plaques to promote Aβ clearance, thus slowing the progression of AD [13,22,28,29,30]. In more advanced stages, the chronic activation of microglia and astrocytes turns disadvantageous, as they lead to the constant overproduction of inflammatory mediators, resulting in prolonged neuroinflammation. Activated microglia have a negative effect on neurogenesis as well [13]. Studies showed that the induction of inflammation processes by bacterial lipopolysaccharide (LPS) reduced the amount of BrdU+ and DCX+ cells and increased the number of microglia in DG [31,32,33]. Others reported that intracerebroventricular (ICV) injections of streptozotocin (STZ) significantly decreased the number of BrdU+ and DCX+ cells and increased neuroinflammation in DG [34,35]. These findings support the connection between neuroinflammation and decreased neurogenesis in AD models. Microglia and astrocytes may serve as early biomarkers of AD, and the elements of the inflammatory system might serve as potential therapeutic targets against the disease [13,28,29,30,36].

The protein Fe65, which is also known as amyloid beta A4 precursor protein-binding family B member 1 (APBB1), belongs to a family of multidomain adaptor proteins. It plays a crucial role in APP processing and trafficking, Aβ production, synapse formation, synaptic plasticity and in the learning process [37,38,39,40,41]. Transcriptionally active AICD is produced during the amyloidogenic pathway only, and can bind to Fe65 and co-binds to Tip60, forming a protein complex [37,40,41]. The complex translocates to the nucleus, where it regulates the expression of various genes (APP, BACE1, ADAM10 and GSK3β), and thereby inhibits proliferation [20,42,43,44,45,46]. In a study, overexpressed AICD bound to Fe65 negatively influenced neurogenesis in the FeCγ25 mouse strain (which co-expressed AICD and human Fe65), and the number of BrdU+ and DCX+ cells were significantly reduced as well [47]. Another study demonstrated that TAG1, the functional ligand of APP, supported AICD release, and neurogenesis was negatively regulated by Fe65 via the TAG1-APP pathway [48]. In our previous study, the peptide P33 (synthesized in-house) was also found to bind to Fe65. We have demonstrated that P33/Fe65 binding decreased the quantity of the Fe65/APP complex, leading to altered APP processing and thereby diminished Aβ production. Moreover, we have revealed that P33 treatment has a favorable effect on memory functions, learning ability and ameliorates inflammatory processes in 9-month-old APP_Swe_/PS1_dE9_ mice [49].

Several transgenic mouse strains were generated to model FAD and investigate the development of AD pathology [8,12,16,50]. Among these, the APP_Swe_/PS1_dE9_ (APP/PS1, Tg) model is a transgenic animal strain demonstrating Aβ pathology with histological features similar to those found in humans [16,25,26,43,51,52]. The APP_Swe_/PS1_dE9_ line harbors two mutations, whereby these animals overexpress the chimeric mouse/human amyloid precursor protein (Mo/HuAPP695_Swe_) with a Swedish mutation (K595N/M596L), and a human presenilin-1 with a deletion of exon 9 (PS1_dE9_) [53,54]. Although several papers discuss the alteration of neurogenesis in the APP_Swe_/PS1_dE9_ strain, to the best of our knowledge, none of these studies have examined the progression of neurogenesis and gliosis throughout the lifespan of these transgenic animals.

In our current study, APP_Swe_/PS1_dE9_ transgenic and C57BL/6J control mice were examined by histological methods from 1 to 12 months and at 18 months of age. Temporal changes in hippocampal neurogenesis, regarding both proliferation and differentiation processes, followed. In parallel, the activation of microglia and astrocytes was also assessed. Besides the age-related changes in the levels of APP, the soluble oligomeric forms of Aβ_1-42,_ C99/C83 ratio and the density of Aβ plaques were also measured. Based on these findings, a related study using 3-month-old APP/PS1 mice was executed to examine the effects of long-term (6-month) treatment with P33 on hippocampal neurogenesis associated with APP-Aβ pathology in this AD model.

## 2. Results

### 2.1. Hippocampal Neurogenesis Is Impaired by Aging in APP/PS1 and Wild-Type Mice

**Figure 1:**
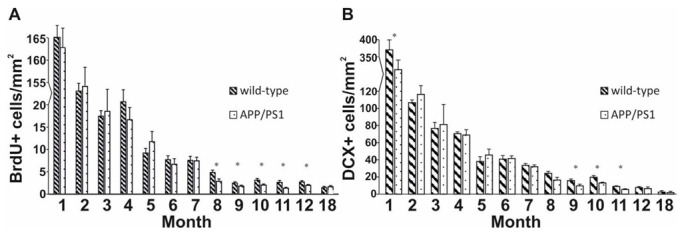
Quantitative results for BrdU and DCX stainings in the dentate gyrus. (**A**) Significant differences were observed among the groups from 8 to 12 months of age: transgenic mice had significantly fewer BrdU+ stem cells than controls. (**B**) DCX+ cells of the DG were analyzed in wild-type and APP/PS1 mice every month. Independent *t*-tests revealed significant differences in the 9-, 10- and 11-month-old age groups. Data represent mean ± SEM * indicates a significant between-group difference (*p* < 0.05).

To characterize the first stage of neurogenesis, we stained dividing stem cells with 5-Bromo-2′-Deoxyuridine (BrdU) [55]. In the first month of life, the number of BrdU+ cells increased in both the Tg and WT groups, while between months 2 and 7 no significant between-group differences in cell proliferation were detected. From months 8 to 12, significant reductions in the number of BrdU+ cells were revealed in APP/PS1 mice compared to age-matched WT controls (Figure 1A, independent sample *t*-test: t_8_ = 2.529 *p* = 0.039, t_8_ = 2.961 *p* = 0.018, t_8_ = 2.329 *p* = 0.048, t_8_ = 2.843 *p* = 0.022, t_8_ = 2.533 *p* = 0.044, 8 to 12 months, respectively). At 18 months of age, no significant differences in BrdU+ cell density were detected between the groups (t_8_ = −0.545 *p* = 0.601). Along with aging, the number of BrdU+ cells continuously declined both in APP/PS1 and WT mice. A remarkably low density of stem cells (<5 number/mm^2^) was detected from the eighth month of life in all age groups. For representative images, see Supplement (Figure S1A).

To examine the process of differentiation during adult neurogenesis, we labelled immature neurons with a doublecortin (DCX) antibody [55]. The same pattern as the one observed in the longitudinal examination of stem cells was detected: a continuous decrease in differentiating cells was evident along with aging. In the first month of life, a prominent cell density was detected in both groups. Moreover, at 1 month of age a significant difference in the densities of immature neurons was detected between transgenic and control mice (Figure 1B, independent sample *t*-test: t_8_ = 2.330 *p* = 0.048). However, from months 2 to 8, no significant differences were observed between the groups. In the 9- to 11-month-old age groups, a significant reduction in the densities of immature neurons was evident in transgenic mice compared to WT animals (independent sample *t*-test: t_8_ = 3.103 *p* = 0.021, t_8_ = 3.138 *p* = 0.014, t_8_ = 2.947 *p* = 0.022, 9 to 11 months, respectively). Exceptionally low densities (<3 number/mm^2^) were detected in 12- and 18-month-old animals, but at these ages cell densities did not differ considerably between the transgenic and WT groups (independent sample *t*-test: t_8_ = 0.538 *p* = 0.610, t_8_ = 0.511 *p* = 0.623, 12 and 18 months, respectively). For representative images, see Supplement (Figure S1B).

The immunohistochemical analysis of neuronal nuclear marker (NeuN) did not confirm any significant differences in cell densities between Tg and WT mice at any age (Supplementary Figure S2).

### 2.2. Neuroinflammation Increases with Age in APP/PS1 Mice

**Figure 2:**
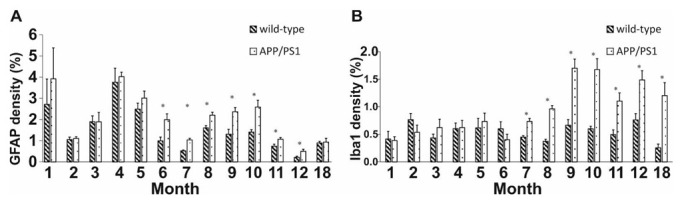
Quantitative results for GFAP and Iba1 stainings in DG. (**A**) Significant differences in the activation of reactive astrocytes were detected between 5- to 11-month-old APP/PS1 mice and age-matched controls. (**B**) Longitudinal alternations of microglial activation in DG. The densities of activated microglia of 7- to 18-month-old Tg mice were significantly higher compared to controls. Data represent mean ± SEM * indicates a significant between-group difference (*p* < 0.05).

To investigate reactive astrocytes which contribute to the neuroinflammatory cascade, we employed GFAP staining. During the first five months of life, there were no significant differences between the groups. In contrast, 6- to 12-month-old APP/PS1 mice were characterized by significantly higher GFAP+ cell densities compared to controls (Figure 2A, independent sample *t*-test: t_8_ = −2.802 *p* = 0.026, t_8_ = −5.441 *p* < 0.001, t_8_ = −4.554 *p* = 0.003, t_8_ = −3.470 *p* = 0.010, t_8_ = −3.832 *p* = 0.006, t_8_ = −2.512 *p* = 0.040, t_8_ = −2.626 *p* = 0.039, 6 to 12 months, respectively). However, once again, the densities of GFAP+ cells were similar in the 18-month-old Tg and WT groups (independent sample *t*-test: t_8_ = 0.176 *p* = 0.605). For representative images, see Supplement (Figure S3A).

To detect activated microglia involved in Aβ clearance and the restoration of a normal brain environment, an Iba1 antibody was used. From 1 to 6 months of age, no differences were observed between the WT and Tg groups. However, the densities of Iba1+ cells increased drastically from 7 to 12 months and at 18 months of age in transgenic mice compared to the WT control (Figure 2B, independent sample *t*-test: t_8_ = −4.743 *p <* 0.001, t_8_ = −8.572 *p* < 0.001, t_8_ = −4.983 *p* = 0.002, t_8_ = −5.315 *p <* 0.001, t_8_ = −7.518 *p* = 0.008, t_8_ = −3.565 *p* = 0.012, t_8_ = 0.003 *p* = 0.013, 7 to 12 and 18 months, respectively). For representative images, see Supplement (Figure S3B).

### 2.3. Age-Related Modulation of APP Processing Pathways in APP/PS1 Mice

To assess the association between neurogenesis and the development of AD, we examined the changes in APP processing pathways by analyzing the level of APP, the C99/C83 ratio by Western blot (WB), the quantity of soluble Aβ_1-42,_ by enzyme-linked immunosorbent assay (ELISA) and the Aβ plaque density by immunohistochemical analyses.

**Figure 3:**
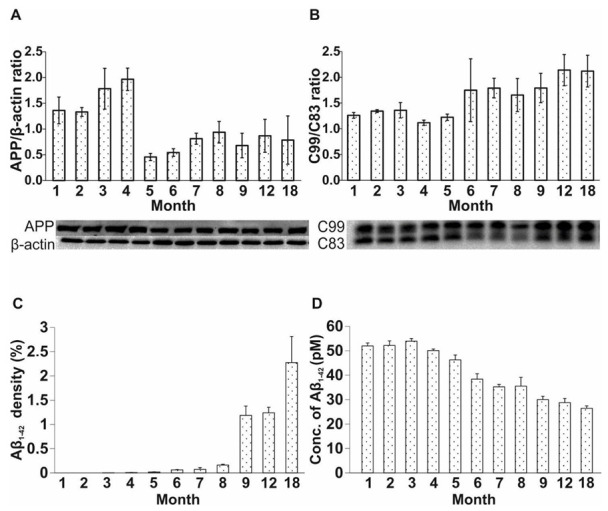
Measurement of APP, C99/C83 ratio by WB, soluble Aβ_1-42_ by ELISA and plaque density by immunohistochemistry. (**A**) APP level was elevated in APP/PS1 animals from months 1 to 4, then decreased in 5- to 18-month-old mice. (**B**) C99/C83 ratio was elevated in Tg animals, starting from 5 months of age. (**C**) Aβ plaques appeared in APP/PS1 mice at 4 months of age. (**D**) The concentration of soluble Aβ_1-42_ decreased in APP/PS1 mice in the 5- to 18-month-old age groups. Data represent mean ± SEM.

The amount of APP may be an early biomarker of AD [23,56,57,58]. The APP level was elevated in Tg mice from 1 to 4 months of age, while it decreased between months 5 and 18 (one-way ANOVA F = 18.279, *p* < 0.001; Fisher’s LSD post hoc tests: 1st month: p4 = 0.008; p5 < 0.001; p6 < 0.001; p7 < 0.001; p8 = 0.002; p9 < 0.001; p12 < 0.001; p18 < 0.001; second month: p5 < 0.001; p6 < 0.001; p7 < 0.001; p8 < 0.001; p9 < 0.001; p12 < 0.001; p18 < 0.001; third month: p5 < 0.001; p6 < 0.001; p7 < 0.001; p8 < 0.001; p9 < 0.001; p12 < 0.001; p18 < 0.001; fourth month: p1 = 0.008; p5 < 0.001; p6 < 0.001; p7 < 0.001; p8 < 0.001; p9 < 0.001; p12 < 0.001; p18 < 0.001) (Figure 3A).

C83 is produced during the non-amyloidogenic processing of APP, while C99 is formed along the amyloidogenic pathway. Thus, the C99/C83 ratio represents the enzymatically regulated balance between these two pathways. During the first five months of age, no difference in the C99/C83 ratio was detected between the groups. From months 6 to 18, an appreciable difference in the C99/C83 ratio became evident for the APP/PS1 group, suggesting an incipient predominance of the amyloidogenic pathway from the sixth month (one-way ANOVA F = 11.499, *p* < 0.001; Fisher’s LSD post hoc tests: first month: p6 < 0.001; p7 < 0.001; p8 = 0.013; p9 < 0.001; p12 < 0.001; p18 < 0.001; second month: p6 < 0.001; p7 = 0.004; p8 = 0.040; p9 < 0.001; p12 < 0.001; p18 < 0.001; third month: p6 < 0.001; p7 = 0.003; p8 = 0.033; p9 < 0.001; p12 < 0.001; p18 < 0.001; fourth month: p6 < 0.001; p7 < 0.001; p8 < 0.001; p9 < 0.001; p12 < 0.001; p18 < 0.001; fifth month: p6 < 0.001; p7 < 0.001; p8 = 0.003; p9 < 0.001; p12 < 0.001; p18 < 0.001) (Figure 3B).

The density of Aβ plaques may correlate with the development of AD in Tg mice. In our experiment, the plaques appeared in the cortical and hippocampal regions in 4-month-old APP/PS1 mice. The amount of Aβ plaques progressively increased with age (one-way ANOVA F = 7.336, *p* < 0.001; Fisher’s LSD post hoc tests: first month: p9 = 0.015; p12 = 0.019; p18 < 0.001; second month: p9 = 0.015; p12 = 0.019; p18 < 0.001; third month: p9 = 0.015; p12 = 0.019; p18 < 0.001; fourth month: p9 = 0.016; p12 = 0.020; p18 < 0.001; fifth month: p9 = 0.017; p12 = 0.021; p18 < 0.001; sixth month: p9 = 0.020; p12 = 0.025; p18 < 0.001; seventh month: p9 = 0.022; p12 = 0.026; p18 < 0.001; eighth month: p9 = 0.032; p12 = 0.038; p18 < 0.001) (Figure 3C). For representative images, see Supplement (Figure S4).

In parallel with the immunohistochemical studies, we examined age-related changes in the levels of soluble Aβ_1-42_ in APP/PS1 mice, using a commercially available ELISA kit. Elevated levels of soluble Aβ_1-42_ were detected in the first 4 months of life, whereas the amount of soluble Aβ_1-42_ strongly decreased in 5- to 18-month-old mice (one-way ANOVA F = 318.481, *p* < 0.001; Fisher’s LSD post hoc tests: first month: p3 = 0.002; p5 < 0.001; p6 < 0.001; p7 < 0.001; p8 < 0.001; p9 < 0.001; p12 < 0.001; p18 < 0.001; second month: p4 = 0.002; p5 < 0.001; p6 < 0.001; p7 < 0.001; p8 < 0.001; p9 < 0.001; p12 < 0.001; p18 < 0.001; third month: p1 = 0.002; p4 < 0.001; p5 < 0.001; p6 < 0.001; p7 < 0.001; p8 < 0.001; p9 < 0.001; p12 < 0.001; p18 = < 0.001; fourth month: p2 < 0.001; p3 < 0.001; p4 < 0.001; p5 < 0.001; p6 < 0.001 p7 < 0.001; p8 < 0.001; p9 < 0.001; p12 < 0.001; p18 < 0.001) (Figure 3D).

### 2.4. Elongated Treatment with Pentapeptide P33 Improves Neurogenesis in APP/PS1 Mice

**Figure 4:**
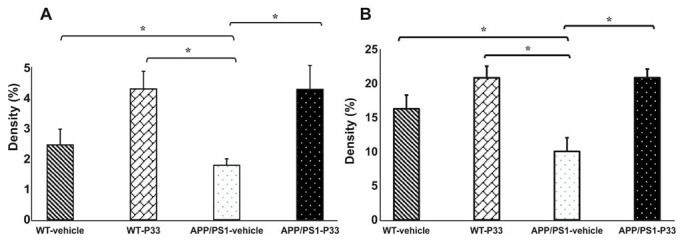
Quantitative results for BrdU and DCX stainings in DG of the animals. (**A**) Significant differences were observed among the groups in the densities of dividing cells (*p* = 0.026). Decreased BrdU+ cell density was detected in the vehicle-treated APP/PS1 group compared to the WT-vehicle (*p* = 0.030), the WT-P33 (*p* = 0.008) and the APP/PS1-P33 groups *(p* = 0.009). (**B**) Results for DCX immunostaining. A significantly lower level of immature neuron density (*p <* 0.001) was detected in vehicle-treated APP/PS1 mice compared to all other groups (vs. WT-vehicle: *p* = 0.028, vs. WT-P33: *p* < 0.001, vs. APP/PS1-P33: *p* < 0.001). Data represent mean ± SEM * indicates a significant difference (*p* < 0.05).

Our previous research indicated that P33-treatment had a beneficial effect on memory, learning abilities and inflammatory processes in APP/PS1 animals [49]. Thus, in a separate experiment, we assessed the effect of elongated P33-treatment on the formation of dividing stem cells and immature neurons in APP/PS1 mice, between 3 and 9 months of age. The WT and APP/PS1 animals were divided into four groups (WT vehicle-treated (physiological saline), WT P33-treated, APP/PS1 vehicle-treated and APP/PS1 P33-treated), and the effects of 6 months of P33 treatment on neurogenesis was examined by monitoring two key markers, namely BrdU and DCX. BrdU+ cell density was found to be significantly lower in vehicle-treated APP/PS1 mice compared to the WT-vehicle and WT-P33 groups (Figure 4A, one-way ANOVA F = 3.845, *p* = 0.026; Fisher’s LSD post hoc tests: APP/PS1-vehicle vs. WT-vehicle *p* = 0.030, vs. WT-P33 *p* = 0.008). In contrast, the density of BrdU+ cells increased in the APP/PS1-P33 group compared to the APP/PS1-vehicle control (*p* = 0.009).

We also measured the density of immature cells in all animal groups. The density of DCX+ cells in vehicle-treated APP/PS1 mice was significantly lower compared to the WT-vehicle and WT-P33 groups (Figure 4B, one-way ANOVA F = 8.939, *p <* 0.001; Fisher’s LSD post hoc tests: APP/PS1-vehicle vs. WT-vehicle *p* = 0.028, vs. WT-P33 *p* < 0.001). Treatment with P33 enhanced the density of immature cells in APP/PS1-P33 mice compared to the vehicle-treated APP/PS1 controls (*p* < 0.001). For representative images, see Supplement (Figure S5).

## 3. Discussion

It is widely accepted that neurogenesis plays an important role in the development and maintenance of memory and learning functions [59,60,61]. Previous experiments have shown that neurogenesis declines during normal aging, as well as in neurodegenerative diseases, including AD [13,15,62].

To the best of our knowledge, we are the first group to study the long-term associations between neurogenesis, neuroinflammation and AD in a transgenic mouse model of AD, using 1- to 12- and 18-month-old wild-type (C57BL/6J) and APP/PS1 transgenic mice. In addition, long-term (6-month) treatment with a peptide-like neuromodulator agent (P33, designed and synthesized in-house) was tested in APP/PS1 animals, starting at 3 months of age, in order to investigate its effect on neurogenesis.

We aimed to compare the processes of hippocampal neurogenesis and neuroinflammation in C57BL/6J vs. APP/PS1 mice, to evaluate their relationship with age and AD-like pathology. Our further goal was to investigate the effect of P33 on neurogenesis in both mouse strains, as this agent has previously been demonstrated to be effective against neuroinflammation induced by Aβ pathology in our former study [49].

Studies report that signs of decreased adult hippocampal neurogenesis are evident in AD, which may cause cognitive dysfunction. Mapping the molecular processes involved in altered neurogenesis would be essential to clarify whether it precedes the characteristic appearance of AD pathology, or rather if it is a consequence of that [13,22,63]. To elucidate this issue, many groups have investigated the relationship between AD and neurogenesis in several FAD transgenic models; however, published studies have yielded controversial results (Table 1). Both an increase [64,65,66,67] and a decrease [25,26,52,68,69,70,71,72] in the levels of DG markers typical of adult neurogenesis are reported for AD. These discrepancies are likely to be explained by different experimental designs, such as utilizing different mouse models and different time scales for the examination of neurogenesis and AD, as well as a variety of selected neurogenesis markers tested.

As it is seen from Table 1, our findings also contradict published experimental data in several aspects. An analysis of the literature’s data has revealed that direct comparison is difficult due to different experimental setups. The frequency of injections, combined with different time points for sacrificing the experimental animals (i.e., different survival times) can result in large differences and complicates comparability [75]. A common feature of cited articles is that the same exogenous marker, BrdU, was injected intraperitoneally to detect stem cells. BrdU is a thymidine analog that can be incorporated into DNA during the S phase of cell cycle [55]. Regarding the administered dose of BrdU, the majority of the authors used 50 mg kg^−1^ [25,36,52,71,73], while two groups injected 100 mg kg^−1^ [26,63]. Depending on the dose of BrdU, divergent cell groups in the S phase of the cell cycle may be detected [76]. Besides dosing, the injection protocol of BrdU is also an important factor. The frequency of BrdU administration is quite diverse in the literature: some groups applied the injection for 1 [69], 3 [25,26,52], 5 [73] or 12 days [71] once a day, while one group applied a vaccination protocol of several times a day [36]. In our experiments, mice were treated with BrdU (100 mg kg^−1^) once daily for 6 days, to label the largest possible amount of proliferating cells [77]. In addition, the timing of sacrificing the experimental animals also differs among published studies. Zeng et al. completed sacrificing within a few hours post-treatment [63], while others sacrificed the animals 1 to 3 days after the last BrdU injection [25,36,52,69]. Verret et al. and Unger et al. conducted extraordinarily long-term studies, and sacrificed the animals 1 month after the last BrdU administration [71,73]. In our experiment, mice were sacrificed 14 days after the last BrdU injection. The dose and injection protocol, as well as the timing of sacrificing the animals, were chosen to optimally study the long-term survival of stem cells. Proliferation can be examined within a few hours or one day after the BrdU injection only, while studying the long-term survival of stem cells requires a longer follow-up period [78].

In WT and Tg mice, decreasing trends in the densities of both BrdU+ and DCX+ cells were observed with aging. The densities of stem cells and immature neurons were high both in 1-month-old wild-type and Tg mice, which could be explained by the superior neuroplasticity of the young brain [79]. The density of BrdU+ and DCX+ cells showed a gradual decrease from 2 months of age. At older ages (8 to 12 months), significant differences were detected between the two strains in both neurogenesis markers. Similarly, other research groups also reported differences in the densities of BrdU+ [25,52] and DCX+ cells [52,69] in 9- and 10-month-old mice, while some groups also reported significant differences in young animals (2 to 5 months of age, BrdU: [26,36,69,73], DCX: [26,36]). These mixed results may result from using APP/PS1 mice with different genetic backgrounds (>10 vs. 0 generations in the C57BL/6J strain) and varying methods [25,73].

Consistent with the results published by Liu et al. [36] and Zhang et al. [74], no changes were observed in the densities of mature neurons (NeuN), either in the WT or in the Tg groups in our study.

In the so-called APP trafficking, which involves the expression, migration and enzymatic processing of APP, the forming products of both the amyloidogenic and non-amyloidogenic pathways play crucial roles in neurogenesis as well. In AD, the amyloidogenic pathway predominates, which is controlled by β-site APP cleaving enzyme 1 (BACE1). Enzymatic cleavage of APP by BACE1 yields C99 and Aβ fragments, contributing to the formation of Aβ plaques. APP level was found to be high in the first 4 months of life, and decreased from 5 months of age, while changes in the C99/C83 ratio followed a reverse trajectory. Demars et al. measured high APP protein levels in 2-month-old APP/PS1 animals [26], which agrees with our findings. Previous studies have shown that both the BACE1 protein level [80] and the enzymatic activity are elevated [81], and BACE1 is accumulated around plaques in AD models, independent of age [80,82,83]. We assume that changes in the amounts of products formed in the amyloidogenic pathway may support the hypothesis that the enhanced expression and/or activity of BACE1 is likely to contribute to the cleavage of APP, resulting in reduced APP and elevated C99 levels in Tg animals.

Our assumption is further supported by the results regarding soluble Aβ_1-42_, which had a high concentration in the first 4 months of life, and started to decrease from the age of 5 months. In parallel with this finding, plaques could be detected from the age of 4 months in the brain of transgenic mice. The reduction in the level of soluble oligomeric Aβ_1-42_ may be explained by the fact that soluble Aβ_1-42_ is re-organized from the oligomeric state to form fibrillar aggregates, manifesting as plaques [84,85,86].

According to the literature, the appearance of plaques in APP/PS1 mice can be observed at different ages. Some groups reported that plaques were first detected in young animals of 3 [29,39] and 4 months of age [73], accompanied by the first signs of changes in neurogenesis [29,69,73]. One group detected Aβ plaques at 6 months of age, after a detectable decline in neurogenic processes (3 months of age) [36]. Taniuchi et al. recognized plaques earlier (5 months of age) than the significant abnormalities first appeared in neurogenesis (9 months of age) [52]. We detected a relatively late (8 to 9 months) onset of altered neurogenesis in transgenic animals, while Aβ plaques could be identified as early as 4 months of age. Therefore, our results support the hypothesis of early plaque formation appearing before the onset of decline in neurogenesis.

Accumulation of Aβ plaques induces inflammatory responses. Activated microglia and astrocytes play an important role in the clearance step of Aβ phagocytosis [13,29,87,88,89]. We have demonstrated that the densities of GFAP+ (at 6 to 12 months of age) and Iba1+ (at 7 to 18 months of age) cells were significantly increased in Tg animals compared to wild-type controls. An increase in the intensity of neuroinflammation became significant after the observed shift in APP trafficking and processing, as well as after the onset of plaque formation. Based on our findings, intensified inflammatory processes could be responsible for the decline in neurogenesis.

The formation of the Fe65/APP complex strongly affects APP processing. On the other hand, the peptide P33 can bind to Fe65, and thus it has the potential to reduce the formation of the Fe65/APP complex. Consequently, it may favorably interfere with the synthesis of Aβ. In agreement with its advantageous biological activity, P33 was shown to significantly improve spatial memory in APP/PS1 mice, increase the number of dendritic spines and decrease the density of inflammatory markers (GFAP, Iba1) [49]. Furthermore, P33 treatment significantly reduced the density of Aβ plaques and the concentration of soluble Aβ_1-42_ [49]. In the present study, P33 treatment significantly increased the densities of BrdU+ and DCX+ cells in the transgenic mouse model. Interestingly, a similar but insignificant change was observed in the number of BrdU+ cells in WT animals as well, suggesting that P33 may enhance cell division under physiological conditions too. This positive effect may be further supported by the specific action of P33 on APP processing. As the binding of P33 to Fe65 hinders the formation of the Fe65/AICD complex, the amount of the transcriptionally active AICD form (which is typical for the amyloidogenic pathway) may be reduced, and this has a beneficial effect on the declining neurogenesis as well. We may conclude that the modulation of the formation of active AICD/Fe65 by P33 possibly reduces the inflammatory processes and thus stimulates neurogenesis.

In conclusion, our experiments demonstrate that neurogenesis is characterized by an age-dependent decline in both wild-type and APP/PS1 animals, with an additional enhancement of inflammatory processes and AD pathology in the latter. Our findings provide evidence that in Tg mice, the early appearance of plaques, quantitative changes in the levels of certain products of the amyloidogenic pathway, as well as the subsequent development of neuroinflammation may contribute to the physiological decline of neurogenesis with aging. Our data support the hypothesis of other research groups, indicating that the molecular development of AD pathology is a driver of the onset of AD symptoms, as well as of the simultaneous impairment of neurogenic processes, which may manifest in decreased densities of stem cells and immature neurons [25,52].

Since AD pathology can be detected even in young APP/PS1 animals, early and elongated neuromodulator therapy may beneficially affect the natural course of AD. In our study, early and long-lasting treatment with P33, a promising neuroprotective peptide, has successfully reduced the rate of decline in neurogenesis in a mouse model of AD, whereby memory and learning disturbances, as well as the decrease in the number of dendritic spines could be minimized. Therefore, P33 is worth being further investigated as a potential drug candidate that may alleviate the characteristic pathological processes of AD.

## 4. Materials and Methods

### 4.1. Animals

For the longitudinal study, 1- to 12- and 18-month-old male and female WT and APP/PS1 mice (*n* = 208) were used. Thirteen groups of 5 were established (male *n* = 2, female *n* = 3). Our previous investigations have shown that the results for males and females did not differ significantly; thus, the smallest possible number of animals were chosen, and even the higher mortality of males during the long time-frame of the experiments had to be considered. Having many years of experience with APPxPS1 transgenic animals, we reckoned this group number and sex ratio routinely feasible to gain statistically evaluable results. For the P33 experiment, 3-month-old male and female WT and APP/PS1 mice were divided into four groups (5 animals each): WT vehicle-treated (physiological saline), WT P33-treated, APP/PS1 vehicle-treated and APP/PS1 P33-treated. Animals were injected with P33 intraperitoneally in a dose of 5 mg kg^−1^ for five days per week, over a course of six months, as described in [49]. APP/PS1 double transgenic mice were obtained from The Jackson Laboratory (USA), from a colony housed at the Biological Research Centre of the Hungarian Academy of Sciences, by breeding APP/PS1 males with WT females (license number: XVI./1248/2017). The mice were kept in groups under constant temperature (23 ± 0.5 °C), lighting (12–12 h light–dark cycle, lights on at 7 a.m.), and humidity (~50%). Standard mouse chow and tap water were supplied *ad libitum*.

All experiments were performed in accordance with Directive 2010/63/EU of the European Parliament and of the Council of 22 September 2010 on the protection of animals used for scientific purposes, and were approved by the regional Station for Animal Health and Food Control (Csongrád County, Hungary; project identification code: I-74-16/2017, 04.07.2017). The experimental protocols were approved by the National Food Chain Safety and Animal Health Directorate of Csongrad County, Hungary (project licenses: XXVI./3642/2017, XXVI./3643/2017). Formal approval to conduct the experiments was obtained from the Animal Welfare Committee of the University of Szeged.

### 4.2. Immunohistochemistry

All chemicals, except the antibodies (Ab), were purchased from Sigma-Aldrich (St. Louis, MO, USA). To detect stem cells, 5 animals per group were injected intraperitoneally (i.p.) with BrdU (100 mg kg^−1^) once a day on 6 consecutive days.

Two weeks after the last BrdU injection, mice were anesthetized with chloral hydrate (1 mg kg^−1^) and were perfused transcardially with phosphate-buffered saline (PBS), followed by 4% paraformaldehyde (PFA). The methods for sample preparation, collection and 4G8, Iba1 and GFAP immunohistochemical stainings were identical to those previously reported [49].

For BrdU staining, the sections were incubated in 2 M HCl for 2 h at room temperature (RT) to denature DNA. Sections were blocked in a mixture of 8% normal goat serum, 0.3% bovine serum albumin (BSA) and 0.3% Triton X-100 in PBS for 1 h. For DCX labeling, sections were blocked in 0.1% BSA and 0.3% Triton X-100 in PBS for 1 h. Sections were incubated at 4 °C overnight with primary antibodies in the following dilutions: mouse anti-BrdU Ab (1:800, Santa Cruz Biotechnology, Dallas, TX, USA), goat anti-DCX Ab (1:4000, Santa Cruz Biotechnology, Dallas, TX, USA), mouse anti-NeuN Ab (1:500; Merck Millipore, Darmstadt, Germany). For BrdU and NeuN stainings, sections were treated with a polymer-based horseradish peroxidase (HRP)-amplifying system (Super Sensitive™ One-Step Polymer-HRP IHC Detection System, BioGenex, Fremont, CA, USA), according to the manufacturer’s instructions. For DCX labeling, sections were incubated with a biotinylated donkey anti-goat Ab (1:1500; Jackson ImmunoResearch, West Grove, PA, USA) for 90 min. The sections were then rinsed in PBS 3 times, and were incubated with an avidin–biotin complex (VECTASTAIN^®^ Elite ABC-Peroxidase Kit; Vector Laboratories, Burlingame, CA, USA) for DCX staining in 1:500, for 90 min at RT. Peroxidase immunolabeling was developed in 30 min using 0.5 M Tris-HCl buffer (pH 7.7) with 3,3′-diaminobenzidine (DAB, 10 mM) at RT. Sections were mounted with dibutyl phthalate xylene (DPX) onto the slides and were coverslipped.

### 4.3. ELISA

WT and APP/PS1 mice (3 animals per group) were euthanized via decapitation. Their brains were removed, and the tissues containing the HC and the cerebral CTX were quickly dissected and stored at –80 °C until further use. The level of Aβ_1-42_ was measured by ELISA as described in [49].

### 4.4. WB

Animal brains were handled the same way as reported for the ELISA experiments. Quantification of APP and C99/C83 protein ratio was determined by Western blot analysis as described in [49].

### 4.5. Quantification of Immunohistochemistry Data

Slides were scanned by a digital slide scanner (Mirax Midi, 3DHistech Ltd., Budapest, Hungary), equipped with a Pannoramic Viewer 1.15.4, a CaseViewer 2.1 program, and a QuantCenter, HistoQuant module (3DHistech Ltd., Budapest, Hungary). For quantifications, all sections derived from each animal were analyzed (12 slices per animal). In DG and HC, the regions of interest (ROI) were manually outlined. Antibody-positive cell types for all ROIs were counted and quantified. The number of stem cells (BrdU+) and neuroblasts (DCX+) were assessed at the border between GCL and the hilus. The densities (%) of neurons (NeuN+), microglia (Iba1+), astrocytes (GFAP+) and Aβ plaques were calculated by the quantification software. To assess cell densities, we divided the total number of counted cells per animal with the DG/HC area, and presented the results as cells/mm^2^ (BrdU+, DCX+) or percentages (NeuN+, Iba1+, GFAP+, Aβ plaques).

### 4.6. Statistical Analysis

The data obtained from the immunohistochemistry analyses were evaluated with Student’s *t*-test for independent samples. WB and ELISA data were analyzed by one-way ANOVA followed by Fisher’s LSD *post hoc* tests. Data analysis was carried out with the SPSS software (IBM SPSS Statistics 24), and the results were expressed as mean ± (SEM). Statistical significance was set at *p* ≤ 0.05.

## Figures and Tables

**Table 1:** Age-related pathological changes in APP/PS1 mice. Comparison table of our findings to literature data. L: lower than WT or low concentration, L-S: significantly lower than WT, H: higher than WT or high concentration H-S: significantly higher than WT, NC: no change in group difference, -: disappearance, +: appearance, NA: data not available, Ref.: references.

Markers		Age (Months)																
		1	2	3	4	5	6	7	8	9	10	11	12	13	14	15	18	Ref.
BrdU	Our study	L	H	H	L	H	L	L	L-S	L-S	L-S	L-S	L-S	NA	NA	NA	H	
	Ref.	NA	L-S [26]	L-S [36,69,73]	NA	L-S [69], L [52]	NA	NA	NA	L-S [25,52]	L-S [69]	NA	NA	NA	NA	L [69]	NA	[25,26,36,52,69,73]
DCX	Our study	L-S	H	H	L	H	H	L	L	L-S	L-S	L-S	L	NA	NA	NA	L	
	Ref.	NA	L-S [26]	H [69,73], L-S [36]	NA	L [69], H [52]	L-S [36]	NA	NA	L-S [52]	L-S [36,69], L [73]	NA	L-S [36]	H [73]	NA	L-S [69]	NA	[26,36,52,69,73]
NeuN	Our study	NC	NC	NC	NC	NC	NC	NC	NC	NC	NC	NC	NC	NA	NA	NA	NC	
	Ref.	NA	NA	NA	NA	NA	NA	NA	NA	NA	NC [74]	NA	NC [36]	NA	NA	NA	NA	[36,74]
GFAP	Our study	H	H	L	H	H	H-S	H-S	H-S	H-S	H-S	H-S	H-S	NA	NA	NA	H	
	Ref.	NA	NA	L [36]	NA	NA	H-S [36]	NA	NA	NA	H-S [36,74]	NA	H-S [36]	NA	NA	NA	NA	[36,74]
Iba1	Our study	L	L	H	H	L	H	H-S	H-S	H-S	H-S	H-S	H-S	NA	NA	NA	H-S	
	Ref.	NA	NA	H [73], L [36]	NA	NA	H-S [36]	NA	NA	NA	H-S [36,74]	NA	H-S [36]	NA	NA	NA	NA	[36,73,74]
Aβ plaque	Our study	-	-	-	+	+	+	+	+	+	NA	NA	+	NA	NA	NA	+	
	Ref.	NA	NA	− [36], + [29], + [69]	+ [73]	+ [52,69]	+ [36]	+ [52]	NA	+ [25,52]	+ [36,69,73,74]	NA	+ [36]	+ [73]	+ [29]	+ [69]	NA	[25,29,36,52,69,73,74]
soluble Aβ	Our study	+	+	+	+	-	-	-	-	-	NA	NA	-	NA	NA	NA	-	
	Ref.	NA	+ [26]	NA	NA	+ [52,69]	NA	+ [52]	NA	+ [52]	NA	NA	NA	NA	NA	NA	NA	[26,52,69]
APP level	Our study	H	H	H	H	L	L	L	L	L	NA	NA	L	NA	NA	NA	L	
	Ref.	NA	H [26]	NA	NA	NA	NA	NA	NA	NA	H [74]	NA	NA	NA	NA	NA	NA	[26,74]
C99/C83 ratio	Our study	L	L	L	L	L	H	H	H	H	NA	NA	H	NA	NA	NA	H	

## Data Availability

Not applicable.

## Supplementary Materials

The following supporting information can be downloaded at: https://www.mdpi.com/article/10.3390/ijms231810364/s1.

## Abbreviations

AD	Alzheimer’s disease
AICD	APP’s intracellular domain
APP	amyloid precursor protein
APP/PS1 APP_Swe_/PS1_dE9_	double transgenic mice
Aβ	amyloid β
BrdU	5-Bromo-2′-Deoxyuridine
BSA	bovine serum albumin
CTX	cortex
DAB	3,3′-diaminobenzidine
DCX	doublecortin
DG	dentate gyrus
DPX	dibutyl phthalate xylene
ELISA	enzyme-linked immunoassay
FAD	familiar form of Alzheimer’s disease
GCL	granular cell layer
GFAP	glial fibrillary acidic protein
HC	hippocampus
HRP	horseradish peroxidase
ICV	intracerebroventricular
i.p.	intraperitoneal
Iba1	ionized calcium-binding adapter molecule 1
LPS	lipopolysaccharide
NPCs	neural progenitor cells
PBS	phosphate-buffered saline
PFA	paraformaldehyde
PS1	presenilin-1
PS2	presenilin-2
ROI	regions of interest
RT	room temperature
sAPPβ	soluble APPβ
SEM	standard error of the mean
SGZ	subgranular zone
STZ	streptozotocin
SVZ	subventricular zone
WB	Western blot
WT C57BL/6J	wild-type mice
