# Supplementary material for: Examination of Longitudinal Alterations in Alzheimer’s Disease-Related Neurogenesis in an APP/PS1 Transgenic Mouse Model, and the Effects of P33, a Putative Neuroprotective Agent Thereon
Source: Int J Mol Sci. 2022 Sep 8;23(18):10364. doi: 10.3390/ijms231810364 (PMC9499399; doi:10.3390/ijms231810364)
Supplement: Supplementary file 1 [file ijms-23-10364-s001.zip › ijms-1908171-supplementary.pdf]

Supplement

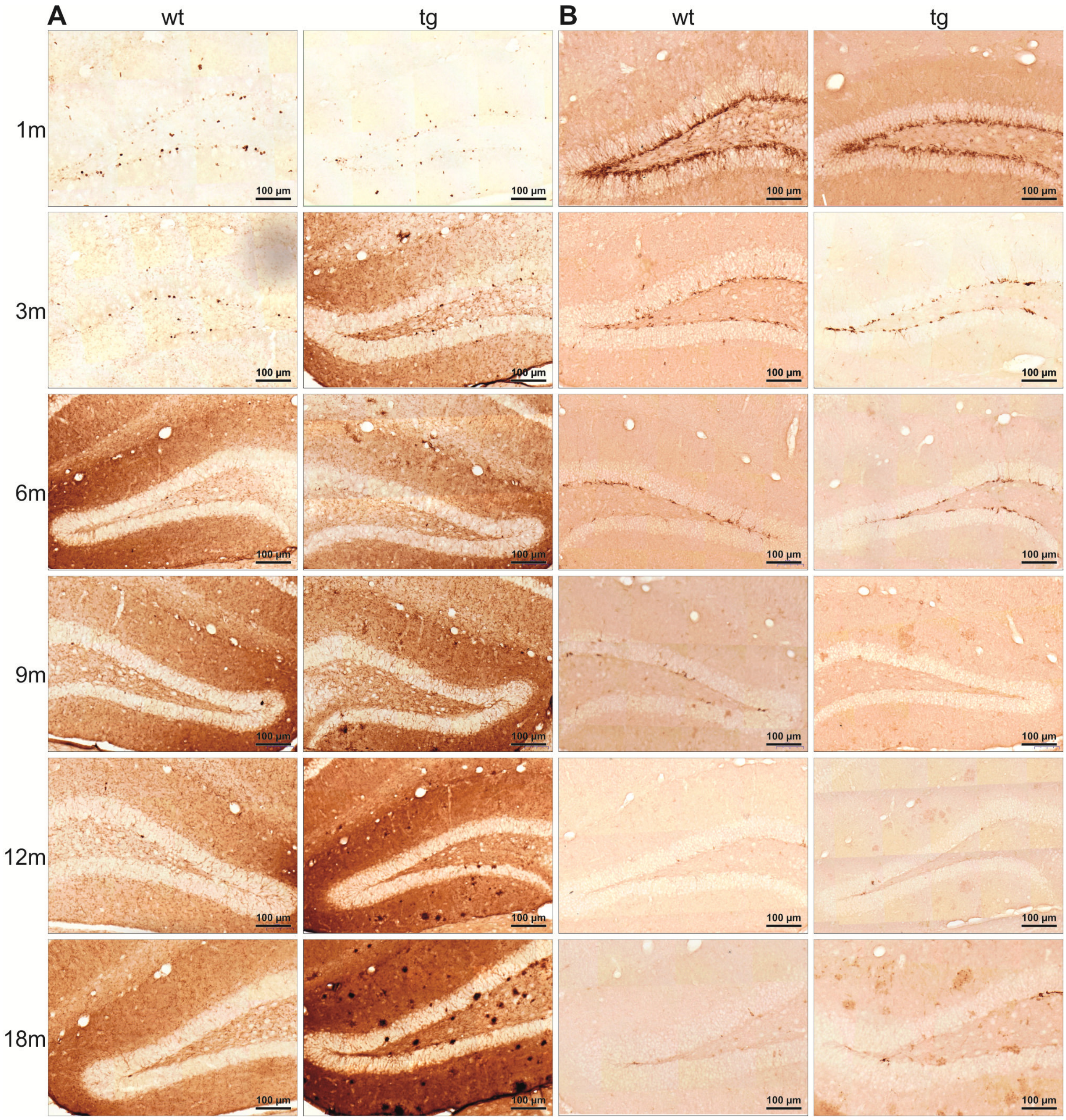

Figure S1: Representative images of BrdU (A) and DCX (B) stainings. Scale bars represent 100 μm.

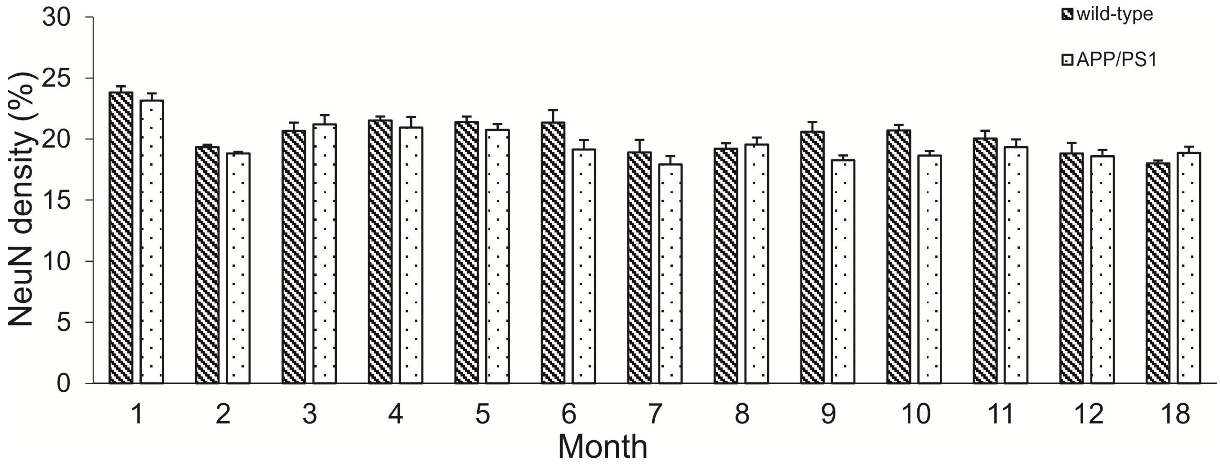

Figure S2: Quantitative results for NeuN staining in dentate gyrus.

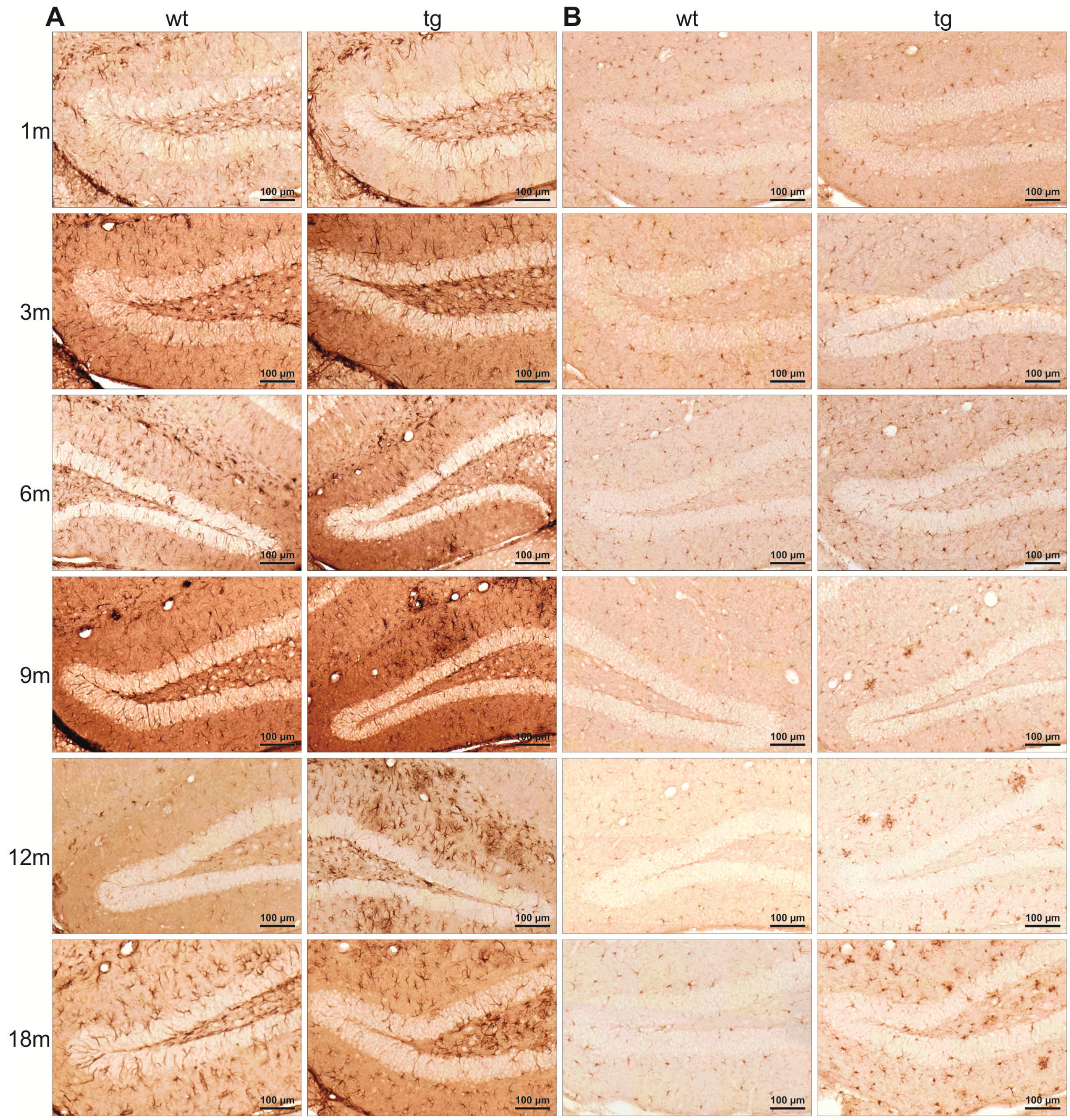

Figure S3: Representative images of GFAP (A) and Iba1 (B) stainings. Scale bars represent 100 μm.

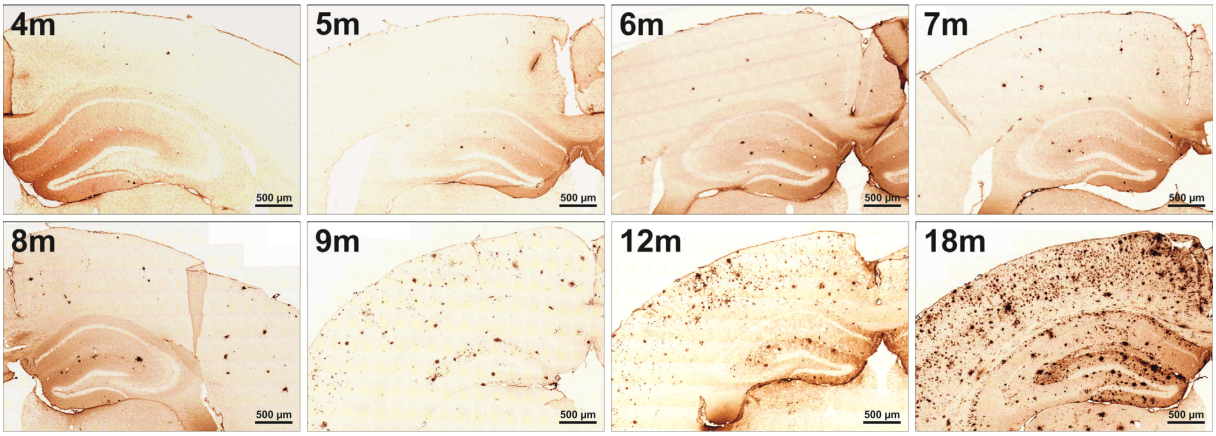

Figure S4: Representative images of 4G8 staining. Scale bars represent 100 μm.

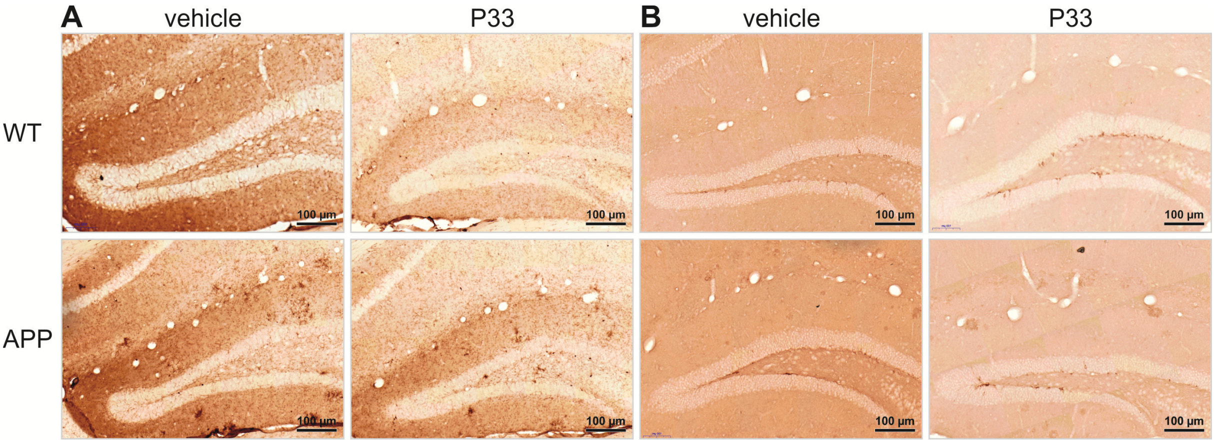

Figure S5: Representative images of BrdU (A) and DCX (B) stainings. Scale bars represent 100 μm.
